# 00003 Volumetric assessment of cervical cancer tumor volume during definitive chemoradiation and the risk of early distant metastasis

**DOI:** 10.1017/cts.2021.688

**Published:** 2021-03-30

**Authors:** Suvidya Lakshmi Pachigolla, Leslie S. Massad, Premal Thaker, David Mutch, Matthew Powel, Farrokh Dehdashti, Barry Siegel, Thomas Mazur, Julie Schwarz, Stephanie Markovina, Perry Grigsby, Alexander Lin

**Affiliations:** Washington University in St. Louis

## Abstract

ABSTRACT IMPACT: This study assesses patient and volumetric risk factors for distant recurrence within 6 months of completion of curative chemoradiation with brachytherapy in locally advanced cervical cancer. OBJECTIVES/GOALS: Initial tumor volume and tumor shrinkage velocity are prognostic of cure and survival after curative chemoradiation (CRT) for cervical cancer. We explored whether local tumor volumetric changes influence time to distant recurrences outside the radiation field. METHODS/STUDY POPULATION: We performed a retrospective cohort study of patients with FIGO Stage IB-IVA cervical cancer treated with curative CRT and brachytherapy at a tertiary academic center with minimum 3 months follow up and standard post-treatment FDG-PET. Patients received 6 weekly fractions of brachytherapy interdigitated with external beam radiation and cisplatin. Tumor volumes were assessed by MRI at brachytherapy planning. Patients who developed distant metastasis were classified as earliest (3-6 months), early (6-24 months) or late (>24 months) following completion of CRT. Absolute and percent decrease in tumor volume for each fraction were calculated with respect to first brachytherapy volume. Fisher’s exact and Mann Whitney-U tests were used for comparison of categorical and continuous variables. RESULTS/ANTICIPATED RESULTS: 143 of 574 (25%) patients developed distant metastasis. Distribution of age, histology, FIGO 2018 stage, primary tumor SUV_max_, treatment length, and pre/post treatment squamous cell carcinoma antigen levels were not associated in each group. Para-aortic lymph metastases were more common in patients with earliest distant recurrence (33% earliest, 26% early, 12% late, p=0.03). Median initial tumor volume in the earliest (n=24), early (n =29) and late (n=9) groups was 57, 28 and 40 mL, respectively (p=0.08); 57 (earliest) vs 30mL (early+late groups), p=0.04. Average mid treatment (fraction 4) and end of treatment (fraction 6) percent shrinkage was 80 (earliest) vs 73 (early+late), p=0.84 and 94 vs 92, p=0.95, respectively. Neither absolute nor percent tumor shrinkage differed between early vs. late groups. DISCUSSION/SIGNIFICANCE OF FINDINGS: Tumor volumetric changes during definitive chemoradiation were not associated with the timing of developing distant metastasis, which is linked to presence of lymph node metastasis and tumor volume at diagnosis.

